# A biological age based on common clinical markers predicts health trajectory and mortality risk in dogs

**DOI:** 10.1007/s11357-024-01352-4

**Published:** 2024-10-01

**Authors:** Sébastien Herzig, Alix Zollinger, Lorane Texari, James A. Holzwarth, Rondo P. Middleton, Yuanlong Pan, Pascal Steiner, Philipp Gut

**Affiliations:** 1 Nestlé Institute of Health Sciences, Nestlé Research, Lausanne, Switzerland; 2 Nestlé Institute of Food Safety and Analytical Sciences, Nestlé Research, Lausanne, Switzerland; 3 Nestlé Research, St. Louis, MO 63164 USA

**Keywords:** Biomarkers of aging, Biological age, Comparative biology, Companion animals, Veterinary care, Calorie restriction

## Abstract

Predicting aging trajectories through biomarkers of biological aging can guide interventions that optimize healthy lifespan in humans and companion animals. Differences in physiology, genetics, nutrition, and lifestyle limit the generalization of such biomarkers and may therefore require species-specific algorithms. Here, we compared correlations of standard clinical blood parameters with survival probability in humans with those of the two most common mammalian companion animals, cats and dogs, and highlighted universal and species-specific relationships. Based on this comparative analysis, we generated and validated an algorithm that predicts biological age in canines using a longitudinal dataset with health records, blood count, and clinical chemistry from 829 dogs spanning over 12 years. Positive deviations of biological from chronological age (AgeDev) measured by this composite score significantly correlated with a decreased survival probability (hazard ratio = 1.75 per 1 year of AgeDev, *p* = 3.7e − 06). Importantly, in nearly half of the dogs whose biological age was accelerated by more than 1 year, none or only a single individual marker scored outside its respective reference range, suggesting practical applications for the detection of unfavorable health trajectories. Analyzing samples from a unique 14-year life-long diet restriction study, we show that restricted caloric intake lowers biological age, an effect that can be quantified at midlife years before a difference in survival is observed. Thus, a biological age clock based on clinical blood tests predicts the health trajectories of dogs for use in research and veterinary practice.

**Supplementary Information:**

The online version contains supplementary material available at 10.1007/s11357-024-01352-4.

## Introduction

Aging is defined as a progressive decline of cellular and organ functions and is the main risk factor for non-communicable as well as many infectious diseases [1, 2]. The pace of aging is influenced by multiple factors: adherence to healthy exercise routines and balanced nutrition positively correlate with better health, while exposure to pollution, a sedentary lifestyle, and inadequate nutrition, among others, can reduce life expectancy and increase the risk for chronic diseases. Cats and dogs are the most common companion animals that have shared the same living environments with humans for millennia. The co-evolution of diet and lifestyle habits has contributed to the risk of contracting age-related conditions, such as cognitive decline, impaired mobility, and cardiometabolic disorders with similarities to non-communicable diseases of older human adults [3].

Today, several tools exist to serve as proxies to measure age-related health status in pets such as the Feline Senior Care Guidelines [4], the Canine Geriatric Syndrome (CGS) Assessment [5], the Canine Cognitive Dysfunction Rating Scale (CCDR) [6], the Canine Frailty Index (CFI), and the Health‑Related Quality of Life (HRQL) questionnaire [7]. Telomere length has also been shown to correlate with lifespan of dog breeds [8]. These tools provide valuable insights for evaluating the pets’ health but rely on time-consuming assessments that require practitioners familiar with these tests. Thus, these tools are not commonly applied in pets without particular risk profiles causing potential oversight of unfavorable health trajectories during life stages when prevention can have a substantial impact.

Biological age clocks are emerging as promising markers of human aging trajectories that can be quantified through samples such as blood or saliva [9–11]. The underlying algorithms use molecular or clinical data to compute biological age, a score that estimates the overall health status of individuals relative to their chronological age. Several studies have shown that the difference between biological and chronological age is associated with the risk of age-related diseases and mortality [10–15]. Although biological clocks based on DNA methylation patterns have been constructed for dogs [16–18], their applicability is currently limited. The implementation of sampling and analytical workflows in clinical practice and the relatively high cost of methylation analyses are today the main barriers to DNA-based clocks. In contrast, biological age clocks generated from routine clinical laboratories can provide a practical and cost-effective approach to biological age in pets. These clocks calculate biological age through a composite scoring approach in which each parameter contributes to the prediction of survival. Specifically, for humans, these predictors consist of panels of 5 to 20 clinical laboratory analytes, such as creatinine, albumin, glycated hemoglobin A1c (HBA1c), and fasting glucose [15, 19]. A human biological clock trained on clinical blood markers, termed phenoage, has been developed [15] and correlates with health status in humans. The phenoage algorithm is sensitive to longevity interventions such as calorie restriction and is therefore considered a relevant biological age marker [20, 21]. However, the human phenoage uses a set of blood parameters that are not typically measured in canine clinical research or veterinary practice. For example, markers like c-reactive protein (CRP) are not part of the standard serum biochemistry panel in dogs [22]. Thus, an equivalent test specifically adapted to common veterinary laboratory blood panels may provide a valuable tool to predict health trajectories in companion animals.

Here, we use unique large datasets from a pet care center with well-characterized dog (*Canis lupus familiaris*) and cat (*Felis catus*) cohorts for which health parameters, disease outcomes, and survival have been recorded longitudinally to compare the predictive value of clinical chemistry and hematological parameters on survival between these two pet species as well as human and find both universal and species-specific predictors of mortality risk. Based on these results, we focused on developing a clinical blood marker-based composite score for dogs following a computational approach described for the human phenoage clocks [15]. As a first application, we chose to prioritize a biological age clock for dogs based on the availability of longitudinal observational data together with records from an interventional study with well-characterized effects on health and lifespan [23, 24].

## Material and methods

### Animals

All study protocols were approved by the Nestlé Purina Institutional Animal Care and Use Committee. Data were collected through regular veterinary visits, and samples were collected during nutritional intervention studies for 940 dogs and 1044 cats within a pet care center between 2009 and 2021. Data inclusion was restricted to complete blood counts and serum chemistry panels (Table S1). The distribution of each parameter was evaluated to determine normality, and data for alkaline phosphatase, ALT, AST, creatine kinase, bilirubin, creatinine, GGT, triglycerides, and urea nitrogen were log-transformed to correct skewness. To avoid extreme values due to acute conditions or measurement errors, data falling below the 1st percentile or above the 99th percentile were excluded. These steps led to a reduction in the total number of animals with complete data available to 829 dogs and 802 cats.

### Association of individual blood markers and survival

For exploration of the association of specific blood biomarkers and survival, data consisting of animal information (breed, sex, age) as well as complete blood count (CBC) and complete serum chemistry were extracted. Pet status was coded as alive (0) if they were still in the center at the time of data extraction or censored at the date of adoption if they left the center. Pets with a recorded date of death were coded as dead (1).

Dog breed sizes were defined as previously described based on the average weight of the breed [25, 26]. The dogs in this study belonged to either small (less than 10 kg) or medium/large (10–35 kg) sizes. We recorded the biomarkers as time-dependent covariates using time intervals open on the left and closed on the right (i.e., (tstart, tstop]) using the survival R package (v. 3.2–13) [27]. Specifically, the biomarker information corresponds to the start of the interval, and the event (alive or dead) is recorded for the end of the interval.

For human data, we used the third National Health and Nutrition Examination Survey (NHANES III) [28]. We extracted relevant information from NHANES III demographic and laboratory files to obtain age, sex, CBC, and serum chemistry data. All-cause mortality in humans was defined as all deaths except accidents, influenza/pneumonia, and other/unknown causes (based on the Underlying Cause of Death (UCOD) code recorded in the NHANES mortality file), and follow-up was restricted to 10 years after the survey.

Next, we scaled the data of each of the 28 biomarkers, so that each biomarker would be centered on 0 and have a variance of 1, and we fitted a Cox proportional hazard (PH) model for survival. In dogs, we included sex and breed size as covariates. For cats, only the biomarker was included as we observed no effect of sex in the model, and the dataset consisted of only one breed. In humans, we included sex to account for the known differences in survival by gender. We adjusted the *p*-value of each parameter to account for multiple comparisons (Benjamini and Hochberg test). The clustering of the hazard ratio was based on a Euclidian distance matrix, excluding incomplete observations, and visualized using the pheatmap R package.

### Selection of biomarkers for the biological age algorithm

We fitted a LASSO-penalized Cox PH model using the glmnet R package (v4.1–3) [29] on the data and used 20-fold cross-validation to select the penalization parameter lambda. Using this approach, the best performance was observed at log(λ) =  − 5, which retained 10 non-zero parameters. These parameters were used to develop the biological age as described in the following paragraph.

### Generation of biological age algorithm

To generate the biological age algorithm, we fitted a Gompertz distribution on the training set including the 10 selected parameters as well as breed size (small or medium/large) and animal sex as covariates, and the age of the animal as the time variable (model 1). This Gompertz model has the following survival function for the *j*th dog:

$$S1\left({age}_{j},{x}_{j}\right)={e}^{{\frac{-{e}{xb}_{j}}*\left({e}^{\left(\gamma *{age}_{j}\right)} - 1\right)}{\gamma }}$$

where $$xb= \beta$$ + $$\sum (coef *value)$$

We then fitted a second Gompertz distribution that only included breed size and animal sex (model 2), with the following survival function:

$$S2\left({age}_{j},{breed}_{j}\right)={e}^{\frac{-{e}^{(breed*{\beta }_{breed}+{\beta 0}_{breed})}*\left({e}^{({{age}_{j}*\gamma }_{breed})} - 1\right)}{{\gamma }_{breed}}}$$

Through this approach, at a given age, the survival probability of a dog from model 1 [S1(${\text{age}}_{j},{x}_{j}$)], in which the time variable is the dog’s chronological age, should be equal to the survival probability according to model 2 $[S2\left({\text{BioAge}}_{j},{\text{breed}}_{j}\right)]$, where the time variable is the dog’s biological age (BioAge). By solving *S*1 = *S*2, we obtain a formula for BioAge, which depends on the 10 biomarkers, age, and sex, as shown below.

$$phenoage=\text{ln}\left(\frac{{\gamma }_{0}*{e}^{xb}*\{{e}^{\gamma *age}- 1\}}{{e}^{\left\{breed*{\beta }_{breed}\right\}+\left\{sex * {\beta }_{sex}\right\}+ {\beta }_{0}}* \gamma }+1 \right) *\frac{1}{{\gamma }_{0}}$$

where $$xb= \beta$$ +$$\sum (coef *value)$$

This approach is similar to that used to construct the human phenoage [15], except that in our case, the time variable represented the animal age as opposed to the follow-up time, and the blood parameters were used as time-dependent covariates. The parameters of the Gompertz regression model were estimated using the flexsurv R package (v 2.1) [30] and are shown in Fig. S3 and S6. Once the dog’s biological age is estimated, we calculate AgeDev by subtracting chronological age from biological age.

### Calorie restriction study

The dog life-long diet restriction study has been described previously [23]. In brief, the study was a pair-feeding study that enrolled 48 Labrador Retrievers for a life-long intervention starting at 8 weeks of life. Pairs of dogs received either a control diet or a 25% calorie-restricted diet compared to the control formulation. Serum biochemical analyses were obtained in addition to the evaluation of body condition and composition. We used retrospective analysis of past CBC and serum chemistry analyzed throughout the study together with survival records [24].

### Application of human phenoage to dog data

To apply the human phenoage to dog data, we used the published phenoage algorithm from [15] and implemented in the BioAge R package [20]. We kept the set of nine biomarkers from the original phenoage (albumin, creatinine, glucose, c-reactive protein, lymphocyte percent, mean (red) cell volume, red cell distribution width, alkaline phosphatase, white blood cell count, and age). However, because the routine dog laboratory panel did not contain c-reactive protein, lymphocyte percentage, and red cell distribution width, we truncated the formula to ignore these parameters.

### Survival analysis

For survival analysis using AgeDev, associations with survival were assessed through Cox PH using the R survival package [27], while for predictions using Gompertz coefficients (parametric model), we used the flexsurv R package [30]. Age was used as the time variable. For the evaluation of the cutoff point along the range of AgeDev, we applied the method proposed by Contal and O’Quigley [31]. For ROC curves, we used the RisksetROC R package to compute the ROC curve for survival prediction. We considered the outcome as survival at the age of 10 years old predicted by AgeDev measured after the age of 5.

### Statistical analysis

All statistical analysis and data exploration were performed using R [32]. The applied statistical methods are described within the “Results” section and figure legends.

### Data availability

Scripts to analyze biological age based on blood markers and for the computing of AgeDev can be requested from the corresponding authors upon reasonable request.

## Results

### Clinical markers associate differently with mortality in cats, dogs, and humans

We first tested whether changes in individual clinical chemistry and complete blood count (CBC) parameters have distinct predictive values for all-cause mortality when comparing dogs, cats, and humans. To this end, we used longitudinal measurements in 829 dogs and 802 cats collected over a period spanning more than 10 years in a controlled pet care center environment (age range, 2 months–17.7 years; 2 months–20.5 years for dogs and cats, respectively). The dog cohort includes 20 different breeds, with an enrichment for Labrador Retrievers and Beagles, while the cat data consists of Domestic Shorthair cats. Panels of blood markers were analyzed throughout the life of the animals as part of routine veterinary care or because animals were enrolled in nutritional intervention studies. We extracted and curated these data and obtained 5329 and 3475 complete measurements of CBC and serum chemistry in dogs and cats, respectively. Using these datasets, we applied Cox PH models in each species to estimate individual hazard ratios for mortality from all causes (Table 1). In addition, we applied the same approach to analyze a human dataset using the National Health and Nutrition Examination Survey (NHANES), a representative cohort of the United States population that includes clinical parameters as well as mortality records. We restricted the panel to those that are present in all three datasets. Table 1 shows the estimated hazard ratios for all-cause mortality in each species across the marker panel. Significant associations after applying multiple corrections are highlighted in yellow.

**Table 1:**
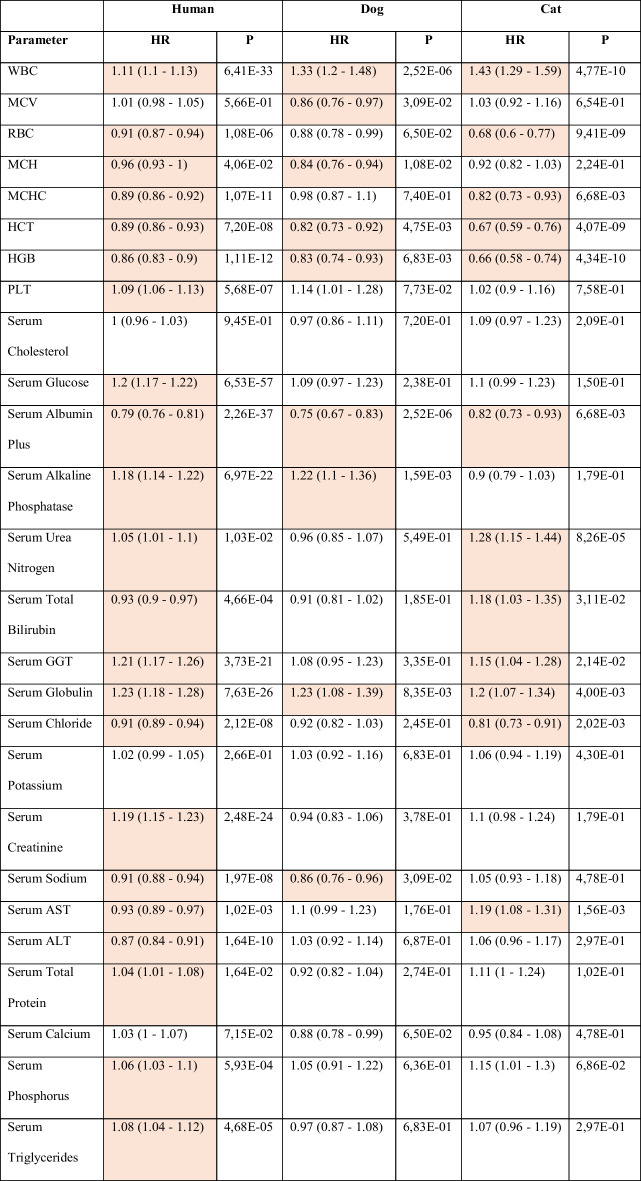
Association of individual parameters (scaled to 1 SD unit) on all-cause mortality in the indicated species

**Fig. 1:**
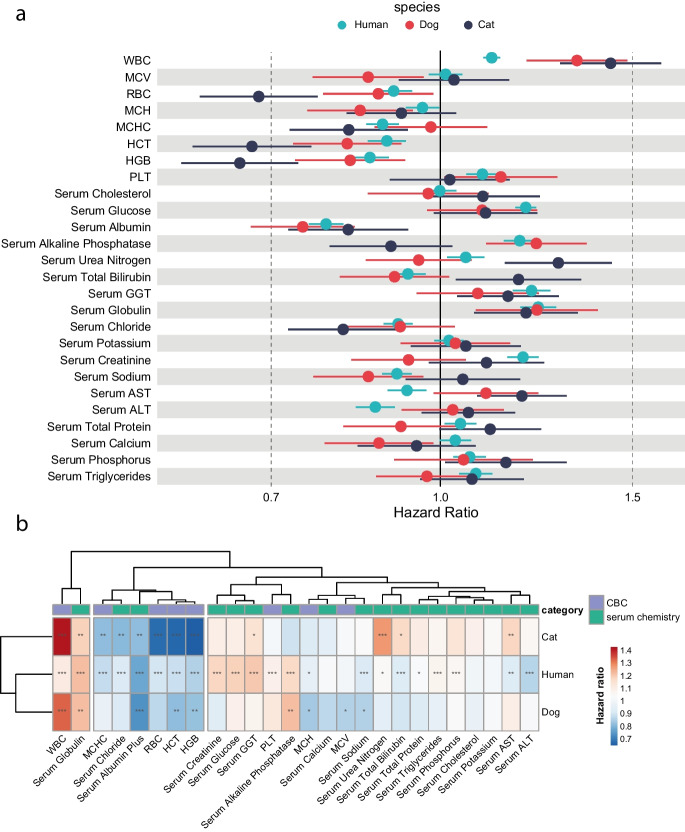
Associations of clinical laboratory markers with survival in cats, dogs, and humans. **a** Comparative analysis of mortality risk and blood parameters in dogs, cats, and humans. The hazard ratio for each parameter was estimated using databases of dogs and cats as well as a publicly available human dataset from the NHANES survey. The hazard ratio for an increase of 1 standard deviation (SD) of each parameter is estimated individually with age as the time variable, and the estimate is shown with 95% confidence interval (CI). For dogs, breed size based on weight and sex was included as covariates, while for cats, the parameters were used as single predictors. For humans, sex was included in the model. **b** Heatmap representation of hazard ratios for all-cause mortality as shown in (a) with significance indicated by asterisks. Species and parameters are clustered by Euclidian distance. *p < 0.05; **p < 0.01; ***p < 0.001. WBC white blood cell count, MCV mean cell volume, RBC red blood cell count, MCH mean corpuscular hemoglobin, MCHC mean corpuscular hemoglobin concentration, HCT hematocrit, HGB hemoglobin, PLT platelet count, GGT gamma-glutamyltransferase, AST aspartate aminotransferase, ALT alanine aminotransferase

Based on this analysis, we identified universal and species-specific predictors of mortality by comparing the markers measured in all three species (Fig. 1a). For example, white blood cell (WBC) count, levels of serum albumin, serum globulins, and hematocrit show similar strength and direction of association in all three species. When clustering these results through Euclidian distance calculation, we observed that humans and dogs showed a closer relationship compared to cats (Fig. 1b). We also identified diverging directionality of effect for some parameters, most notably for urea nitrogen levels, which showed strong associations with mortality in cats but not in dogs (Fig. 1a and b). Thus, analyses of longitudinal pet and human cohorts enabled a comparative approach that showed similar and distinct associations to all-cause mortality in dogs, cats, and humans and confirmed that specific parameters and their weights need to be evaluated in the target species. Beyond these statistical differences in associations to mortality, the availability of markers routinely measured in pets is an important consideration for the selection of parameters as part of a phenoage algorithm. Of note, CRP, lymphocyte percentage, and red cell distribution width (RDW) are part of the original human phenoage algorithm but were not present in the dog routine laboratory analysis in our dataset.

### A clinical blood marker-based clock predicts mortality in dogs

Next, we built on these results to construct an aggregated score as a surrogate for overall health in dogs. To this end, we applied a method previously used to train a human phenotypic age clock [15]. The steps to select, train, and test the algorithm are summarized in Fig. S1. The standard CBC and serum chemistry panel included 28 parameters (Table S1) across the longitudinal dataset. We randomly distributed dogs into training or testing sets using 75% of the dogs for training and the remaining 25% for the testing set. We next used a penalized Cox regression model to select the most meaningful and parsimonious set of biomarkers to predict mortality on survival data stratified by breed size. Breed size was defined by the weight of the breed as detailed in the “Methods” section. Using 20-fold cross-validation and a LASSO-penalized Cox regression, we selected a set of ten parameters that is the most predictive for mortality (Fig. S2a). Based on these results, we combined these ten parameters into a canine phenotypic clock, also referred to as the biological age clock (Table 2). To calculate the biological age, we modeled the data using a Gompertz distribution as previously reported for human phenoage [15]. We incorporated the animal’s breed size and sex in the model to account for the known effects of breed and sex on survival. The resulting biological age formulas and coefficients are shown in Table 2 and Fig. S3.

**Table 2:** Gompertz coefficients of the parameters selected for the canine-specific biological age algorithm

Parameter	Coefficient
White blood cell count (10^3 per µL)	0.0919
Hemoglobin (g/dL)	− 0.00913
Mean cell volume (fL)	− 0.00749
Hematocrit (%)	− 0.0184
Mean corpuscular hemoglobin (pg)	− 0.128
Serum glucose (mg/dL)	0.00917
Serum globulin (g/dL)	0.133
Serum creatine kinase (U/L, ln-transformed)	0.333
Serum albumin (g/dL)	− 0.744
Serum alkaline phosphatase (U/L, ln-transformed)	0.263

See Fig. S3 for full model parameters

*µL* microliter, *IU/L* international units per liter, *U/L* units per liter

**Fig. 2:**
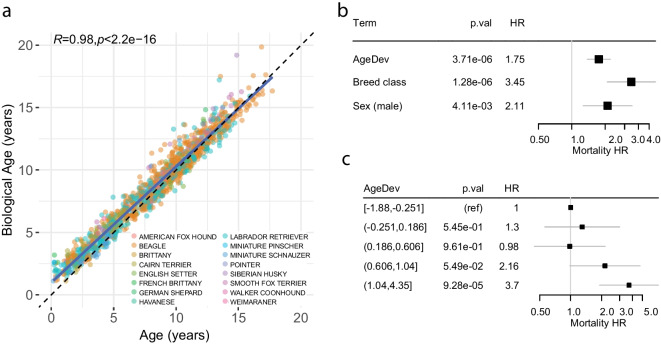
Blood marker-based composite score predicts mortality in dogs. **a** Application of the biological age algorithm to the testing sets of dogs consisting of animals that have not been used to identify the coefficients of the model (training set). The colors of the points indicate different breeds as shown in the legend. **b** Cox PH model of increased AgeDev (defined as the difference between biological age and chronological age) for mortality in dogs. HR indicates a hazard ratio for a 1-year increase in AgeDev. **c** Cox PH model of AgeDev quintiles for mortality in dogs. In **b** and **c**, the estimate is shown with 95% CI. HR = hazard ratio per 1 year of AgeDev

Next, we used the testing set, corresponding to an independent group of 208 dogs not used to train the model, to validate the algorithm. Biological age showed a highly significant Pearson’s correlation with chronological age of 0.98 (*p*-value < 2.2e − 16) (Fig. 2a). A Cox PH model showed that the delta age, hereafter termed age deviation (AgeDev), defined as the difference between biological age and chronological age [10], was associated with a hazard ratio (HR) of 1.75 per year for all-cause mortality independent of breed and sex (*p* = 3.7e − 06). Thus, a 1-year increase in AgeDev is associated with an increased risk of death by 75% irrespective of the dog’s chronological age (Fig. 2b). In comparison, the human phenoage algorithm [15] applied to the available parameters of the dog data set by excluding c-reactive protein, RDW, and lymphocyte counts showed a substantially lower hazard ratio to predict survival when compared to the dog-specific clock. This finding shows that clocks need to be trained with the relevant data for each species to be readily applicable to canine clinical research or veterinary practice (Fig. S4).

We next divided the dog biological age observations of the testing set into quintiles of AgeDev and calculated the hazard ratio for all-cause mortality of each quintile. We observed that compared to the reference group (first quintile), the fourth and fifth quintiles, which have AgeDev range from [+ 0.6 to + 1] and [+ 1 to + 4], respectively, were significantly associated with an increased risk of mortality (Fig. 2c). We assessed AgeDev as a binary measure (low or high) instead of a continuous variable by setting an arbitrary threshold value. To test the possible threshold values, we applied various breakpoints along the range of AgeDev and systematically tested the association of AgeDev as a binary measure (low or high) with mortality. We observed a significant association of mortality when biological age was above the chronological age (threshold for AgeDev = 0), and a marked increase in HR when the cut-off was set between 1 and 1.5 years, consistent with our quintile analysis (Fig. S5). Thus, we consider an AgeDev that is increased by 1 year as a strong indicator for accelerated aging, while more moderate deviations still show substantial hazard ratios for mortality (Fig. 2c).

### Accelerated aging despite largely normal clinical markers

**Fig. 3:**
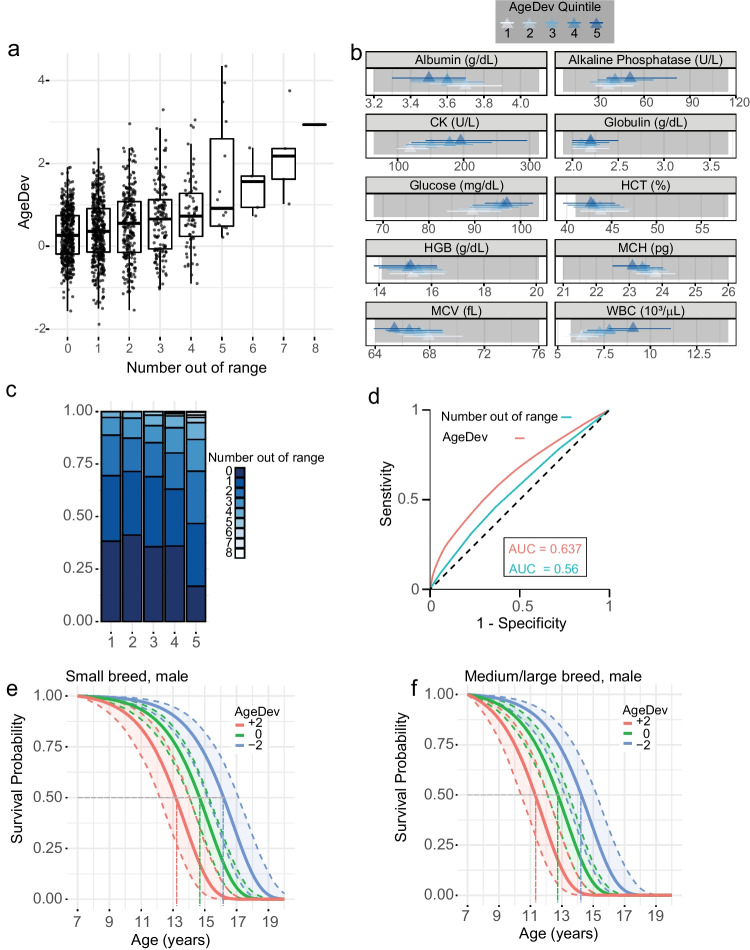
Age acceleration and differences in survival despite largely normal clinical markers. **a** AgeDev stratified by the number of parameters outside of clinically defined normal ranges. Data are represented as boxplots showing the median line and 1st and 3rd quartiles (box) and whiskers extending 1.5 times the interquartile range (IQR) above and below the box. Individual points are overlayed with the boxplot. **b** Representation of the mean value (triangle) and 1st and 3rd quartile (bands) for each parameter in the different quintiles of AgeDev. The grey area represents the normal range for the given parameter. **c** Representation of the number of out-of-range parameters as stacked bars in each quintile of AgeDev. **d** ROC curve for prediction of survival at the age of 10 based on parameters measured after the age of 5. AgeDev or the number of parameters out of range was used in a Cox PH model. **e** and **f** Survival probability of 7-year-old male small (e) or medium/large (f) dogs with different values of AgeDev were predicted using a Gompertz model. A line represents the survival probability with the indicated parameters, and colored bands represent the 95% CI of the prediction. CK creatine kinase, HCT hematocrit, HGG hemoglobin, MCH mean corpuscular hemoglobin, MCV mean cell volume, WBC whole blood cell count

The biological age algorithm developed in this study is based on a panel of routine laboratory tests that each reflect potential changes in organ systems. We, therefore, tested how clinically defined reference ranges (https://www.vet.cornell.edu/animal-health-diagnostic-center/laboratories/clinical-pathology/reference-intervals) of these parameters were reflected in AgeDev. Extreme cases, here defined as dogs with four or more parameters out of the reference range, were associated with an elevated AgeDev in the majority of animals (Fig. 3a). However, we observed a large variability of AgeDev ranging from approximately − 1.75 to + 2 years even in the absence of parameters out of the reference range (Fig. 3a). We analyzed the distribution of each of the 10 parameters of biological age relative to the clinically defined normal range in the five quintiles of AgeDev. The median of each of the ten parameters falls within the clinically defined normal range for all quintiles while the majority of data points of each marker remain within the reference range even in the 5th quintile (Fig. 3b). Notably, the proportion of parameters out of range only changed in the 5th quintile of AgeDev compared to the other quintiles suggesting that accumulation of minor deviations drives biological age acceleration (Fig. 3c).

To further evaluate the quality of biological age-based mortality prediction compared to numerically counting markers with out-of-range values, we compared Cox PH models analysis using Receiver–Operator Curves (ROC) for the prediction of survival at 10 years of age from parameters measured at the age of 5. As shown in Fig. 3d, the area under the ROC curve (AUC) of the prediction based on AgeDev was superior to the prediction considering only the number of parameters out-of-range.

We next fitted a Gompertz regression to the testing set using AgeDev as a predictor (together with breed size and sex) and used this model to represent the predicted survival probability of a 7-year-old male small or medium/large dog at different values of AgeDev: − 2, 0, and + 2 years (Fig. 3e and f). Survival curves differed substantially under these three scenarios, with a gain of almost 3 years in median survival when comparing the difference between dogs that are biologically younger or older at age 7 (− 2 to + 2 AgeDev). Strikingly, in our predictions, lowering the biological age in a medium/large pure breed dog by 2 years would extend predicted median survival close to the average of small pure breed dogs, which at a population level have a higher life expectancy compared to larger breeds [33].

In conclusion, these data show that an aggregated score of ten clinical parameters can provide additional insights related to the health status and can complement clinical cut-offs for individual markers. Small variations within the normal range influence the overall biological age and allow an estimate of the aging trajectories of dogs.

### Long-term calorie restriction lowers biological age in dogs

**Fig. 4:**
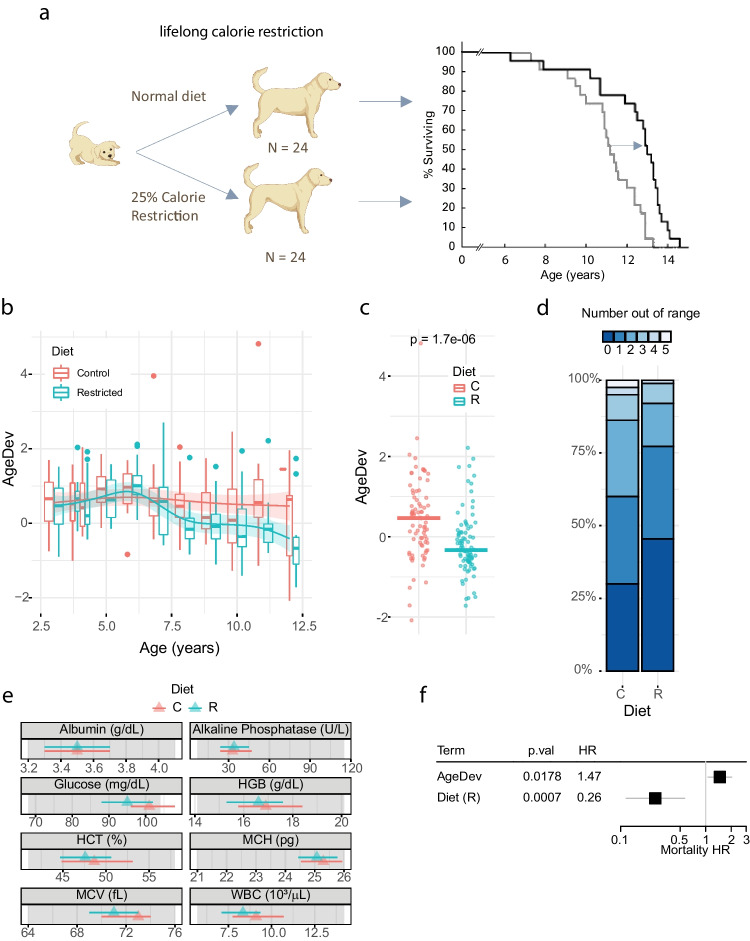
Calorie restriction lowers biological age in dogs. **a** Schematic representation of the Purina Life Span Study adapted from Lawler et al. [23] (created with BioRender.com). **b** Evolution of AgeDev plotted over chronological age. Data are represented as boxplots showing the median line and 1st and 3rd quartiles (box) and whiskers extending 1.5 times the interquartile range (IQR) above and below the box. Data points outside of the whiskers are shown as individual dots. Lines indicate fit from a generalized additive model of AgeDev over time stratified by diet group. **c** AgeDev in dogs older than 7 years. Each dot represents a measure with the line indicating the mean of each group. **d** Percentage of parameters out of range in control (C) or restricted (R) dogs. The colors indicate the number of parameters out of range. **e** Representation of each parameter contributing to biological age shown as mean (triangle) and 1st and 3rd quartiles (band) for each diet group. Grey areas represent clinical reference ranges. **f** Cox PH ratio for AgeDev and diet effects observed in a long-term calorie restriction intervention. The estimate is shown with a 95% CI. C control, R calorie restriction, HR hazard ratio

We next aimed to test whether our canine phenoage clock is sensitive to an established longevity intervention. Calorie restriction (CR) has been widely accepted as a geroprotective dietary pattern in mammalian species [34–36]. Notably, healthy, non-obese people (21–50 years, BMI 22–27 kg/m^2^) that underwent CR over a time frame of 2 years were shown to have a lower biological age, quantified by the human phenoage clock [20, 21] or with recently described principal component analysis (PCA)-based clocks [37]. In dogs, the landmark Purina Life Span Study was performed in the 1990s and showed that feeding dogs to an ideal body condition over a lifetime can delay the onset of clinical signs of age-related chronic diseases and extend the health span in dogs [23, 24]. In the Purina Life Span study, 48 Labrador retrievers were paired at 6 weeks of age and divided into a control and a lean-fed group. Starting at 8 weeks of age, the lean-fed dogs were fed 75% of the calories of the paired control dogs (Fig. 4a) [24]. Lawler et al. [23] showed that feeding to ideal body condition, assessed as a body condition system score between 4 and 6, extended life by 1.8 years and delayed the need for treatment of age-related chronic disease.

We digitally curated clinical chemistry and CBC data from the Purina Life Span study to analyze biological age throughout the 14-year-long intervention period with the aim of testing whether our biological age algorithm is responsive to dietary longevity interventions. Because two parameters of the biological age algorithm, serum creatine kinase, and serum globulin, were missing from the dataset, we retrained the Gompertz model using only the eight available blood parameters using our training set, as described previously (Fig. S6). We observed a similar performance of the modified biological age formula on mortality prediction in the test set (HR = 1.76, *p* = 1.06e − 05).

Next, we applied the modified algorithm to samples from the dogs throughout the 14-year study and compared the results to those from the control-fed dogs. The biological age of the dogs was indistinguishable in the first 7 years of life between groups. However, after 7 years, the lean-fed dogs showed a significantly lower AgeDev compared to control dogs (Fig. 4b and c). Evaluation of the deviation from normal clinical ranges of the blood parameters in senior dogs revealed that the number of parameters out of range also decreased in the lean-fed group, confirming beneficial effects of feeding to ideal body condition (Fig. 4d). When analyzing each parameter of biological age independently, we observed that the lean-fed group had a lower fasting glucose and WBC count, consistent with metabolic and immune benefits that had been observed in the trial [23, 24, 38] (Fig. 4e). Importantly, AgeDev remained an independent predictor of survival independently of diet in a Cox PH analysis (HR = 1.46, *p* = 0.018, *n* = 48 dogs) (Fig. 4f).

Thus, a dog phenotypic age clock can detect the beneficial effects of a dietary intervention on biological age with differences seen already at midlife.

## Discussion

Our comparative biology approach tested associations of blood parameters with mortality outcomes in two common companion animals, dogs, and cats, with those in humans. Several analytes show universal associations with mortality: WBC count, hemoglobin levels, and hematocrit, two indicators of red blood cell quality, as well as serum globulins and serum albumin have the same directionality in all three species. Increases in serum glucose levels are significantly associated with mortality in humans and trend in the same direction in cats and dogs. These results are consistent with previous work that showed that, contrary to rodents, non-human primates (NHP) and humans show a positive association between fasting blood glucose levels and mortality suggesting that across higher mammals changes in glycemic regulation contribute to age progression [39, 40]. In contrast, alkaline phosphatase and sodium levels were associated with survival in dogs and humans but not in cats, while higher bilirubin levels were only associated with survival in cats. Two markers of liver damage, AST and ALT, have negative associations with all-cause mortality in humans consistent with previous reports [41] but not in dogs or cats. Overall, our data suggest that cats exhibit a more distinct pattern relative to those found in humans and dogs. Similarities in organ physiology and nutritional requirements between humans and dogs, which are omnivores compared to the carnivore diet of felines, may explain at least in part this closer relationship of markers in predicting mortality risk.

Following this comparative analysis, we developed and validated a score aggregating individual blood parameters that together robustly predict mortality risk in dogs as a proxy for overall health status. The HR shows a 1.75-fold increased mortality risk per year of accelerated age. In the group of animals between 1 and 4 years of age acceleration compared to their chronological age, this risk further increased to 3.7-fold. These HRs are higher than those reported for biological clocks in humans [11], likely reflecting the faster pace of aging and ultimately shorter life expectancy of dogs compared to that of humans.

A limitation of our study is that the cause of death reported in the dataset is in most cases the result of humane euthanasia to prevent unnecessary suffering of animals with advanced chronic disease. The decision to euthanize is taken by trained veterinarians when no treatment option is available to alleviate pain or otherwise maintain the pet’s quality of life. Our analysis therefore evaluates the association of parameters with advanced terminal disease rather than true survival. However, because the aim is to model and understand a healthy lifespan, we considered morbidity-related euthanasia as an appropriate surrogate for mortality. A further limitation of our approach is that two breeds were overrepresented in the cohort: Beagles, a small breed, and Labrador Retrievers, a medium/large size breed. Although our biological age clock shows a robust prediction of chronological age in several other breeds that were present in smaller numbers in the pet care center, we cannot exclude that the algorithm may need to be adjusted with access to larger numbers of dogs across many breeds.

Our clock uses a relatively small set of input parameters compared to more complex models trained on multi-omics data, which typically use hundreds or thousands of features to predict biological age. While we acknowledge this shortcoming, our approach is pragmatic as it relies on data that are already collected routinely in veterinary practice and that does not require the implementation of new analytical workflows. The availability of markers in clinical practice is a main consideration to enable the broad use of biological age tests in pets, adding to the need to select individual markers based on species-specific associations to mortality. Our biological clock shows a strong hazard ratio for mortality (HR = 1.75), while the accuracy is moderate (AUC = 0.637). This finding is comparable to the performance of biological clocks for humans [42, 43], and is consistent with the concept that such technologies do not qualify as a diagnostic test per se. Their use is proposed to rather complement existing diagnostic tests by raising the indication for deeper health assessments when an accelerated biological age is detected in routine visits.

Beyond the use to detect unfavorable health trajectories, we provide evidence that our algorithm can quantify the efficacy of a dietary intervention on biological age: leveraging data from the Purina Life Span study [23, 24], we show that lean feeding of dogs with a reduced caloric intake of approximately 25% for 14 years robustly lowered biological age. Dietary restriction had the largest effect size on fasting glucose. While this parameter is a strong contributor to the reduction in biological age, WBC count, MCV, and HCT were also responsive to CR and contributed to the effect. These results in dogs are consistent with findings in humans: participants enrolled in CALERIE, a study that investigated an average 15% reduced calorie intake over the course of 2 years in non-obese subjects, showed improved markers of insulin sensitivity and a lower biological age [20–22]. Thus, in higher mammals, clocks based on routine clinical markers can be used to quantify the effects of dietary patterns with reduced calorie intake and provide insights into the individual markers underlying the benefits. Beyond continuous calorie restriction, further studies are needed to identify the most promising dietary regimens to improve biological age. For example, diet patterns under the umbrella of intermittent fasting are also considered an effective means of weight management [44]. Their geroprotective effects are proposed to occur in non-obese subjects independent of weight loss[45] and a fasting-mimicking diet has recently been shown to reduce biological age in humans [46]. Phenotypic clocks as developed here can readily leverage past trials that have available blood count and clinical chemistry data to assess effects on biological age, and thereby add to the understanding of physiological drivers that link specific diet patterns to longevity.

Beyond applications in clinical research, our biological age algorithm may support veterinary applications: In veterinary practice, routine blood tests are used to identify abnormalities in individual blood markers. Typically, concerns are raised when these markers are outside of what is considered the healthy reference range. Our findings show that dogs with AgeDev between 0.6 and 1 year have in 30% of cases none of the analytes outside its reference range. In animals with greater than 1 year of advanced biological age, about 50% of the dogs had none or only one marker out of range. Thus, the composite score approach may be used to flag concerns that may otherwise go unnoticed if only considering reference ranges of individual markers.

Clinical laboratory tests are commonly recommended when dogs are considered mature adults or seniors, the latter being defined as the last 25% of the estimated lifespan until their end-of-life based on population data [47]. Our algorithm detects changes in AgeDev between the groups already at approximately 7 years, a time point before differences in survival can be observed. This work therefore suggests that the ability to quantify biological age around the pets’ midlife may lead to a more individualized approach to determine when a dog should be considered senior and therefore enter frequent health monitoring.

In summary, we present the first clinical blood panel-based biological clock specifically for canines with relevance to veterinary practice or clinical research and that can be readily applied to retrospective or prospective studies that include standard blood laboratory measurements.

## Supplementary Information

Below is the link to the electronic supplementary material.

Supplementary file1 (DOCX 549 KB)
