# Supplementary material for: A biological age based on common clinical markers predicts health trajectory and mortality risk in dogs
Source: GeroScience. 2024 Oct 1;47(1):45–59. doi: 10.1007/s11357-024-01352-4 (PMC11872834; doi:10.1007/s11357-024-01352-4)
Supplement: Supplementary file 1 — Supplementary file1 (DOCX 549 KB) [file 11357_2024_1352_MOESM1_ESM.docx]

**SUPPLEMENTARY INFORMATION**

**GeroScience**

**A biological age based on common clinical markers predicts health trajectory and mortality risk in dogs**

**Sébastien Herzig^1^*, Alix Zollinger^1^, Lorane Texari^2^, James Holzwarth^2^, Rondo P. Middleton^3^, Yuanlong Pan^3^, Pascal Steiner^3^ and Philipp Gut^1^***

# AFFILIATIONS

^1^ Nestlé Institute of Health Sciences, Nestlé Research, Lausanne, Switzerland

^2^ Nestlé Institute of Food Safety and Analytical Sciences, Nestlé Research, Lausanne, Switzerland

^3^ Nestlé Research St. Louis, St. Louis, MO 63164, USA

* Corresponding authors

**CORRESPONDING AUTHORS**

Philipp Gut, ORCID iD: <https://orcid.org/0000-0002-0689-9518>

Email: [philipp.gut@rd.nestle.com](mailto:philipp.gut@rd.nestle.com)

Sébastien Herzig, ORCID ID: [https://orcid.org/0000-0002-9329-2860](https://orcid.org/0000-0002-9329-2860?lang=en)

Email: [sebastien.herzig@rd.nestle.com](mailto:sebastien.herzig@rd.nestle.com)

**Supplementary Figures 1-6**

**Supplementary Table 1**

**Starting dataset:**

28 clinical blood parameters

Petcare center monitoring of 940 dogs and 1044 cats over 12 years

Log transform skewed data

Remove extreme value (retain between 1^st^ and 99^th^ percentile)

829 dogs with 5329 observations

802 cats with 3475 observations

Split 3:1 into train and test sets

LASSO Cox regression on survival

with 20-fold crossvalidation

Selection of biomarkers

COX regression with individual blood marker as predictors

Compute Gompertz distribution with biomarkers. For dogs, include breed class and sex

Construction of Biological Age formula

Calculate Biological Age on training set

Test prognostic value on survival

Validate predictive value of Biological Age in dogs:

Life-long calorie restriction study

- 6 months weight loss intervention study

NHANES survey of US population

CBC and serum chemistry parameters

Dog data

Highlight species-specific predictors of mortality

**Fig S1**

Schematic representation of the workflow of analysis


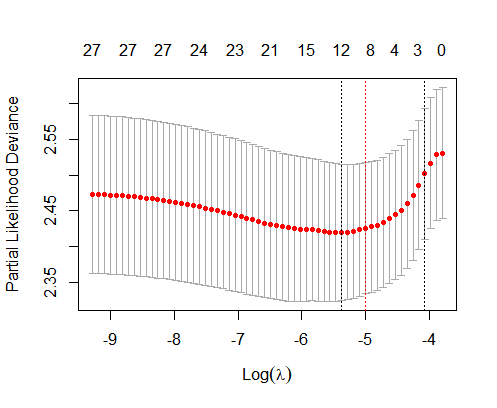


a

b


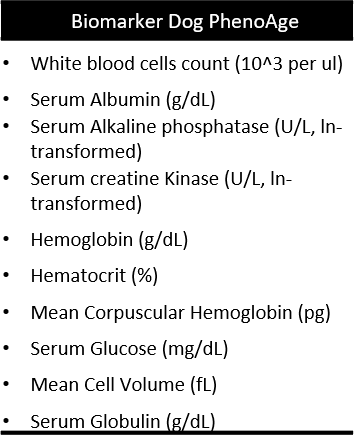


c


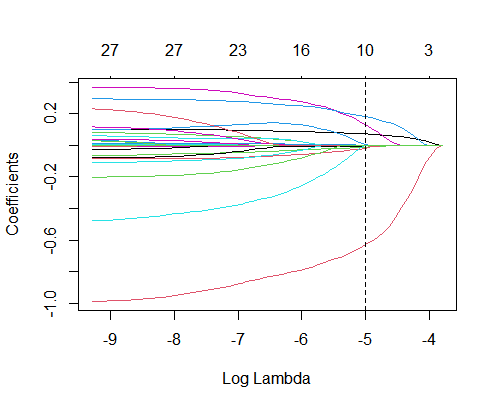


**Fig S2**

**a** LASSO penalized Cox regressions were used to select the optimal number of predictors of mortality in dogs. **b** Selection of the optimal lambda parameter was performed to minimize the number of features while retaining a good fit of data (red dashed line). **c** Retained parameters at the selected lambda threshold.


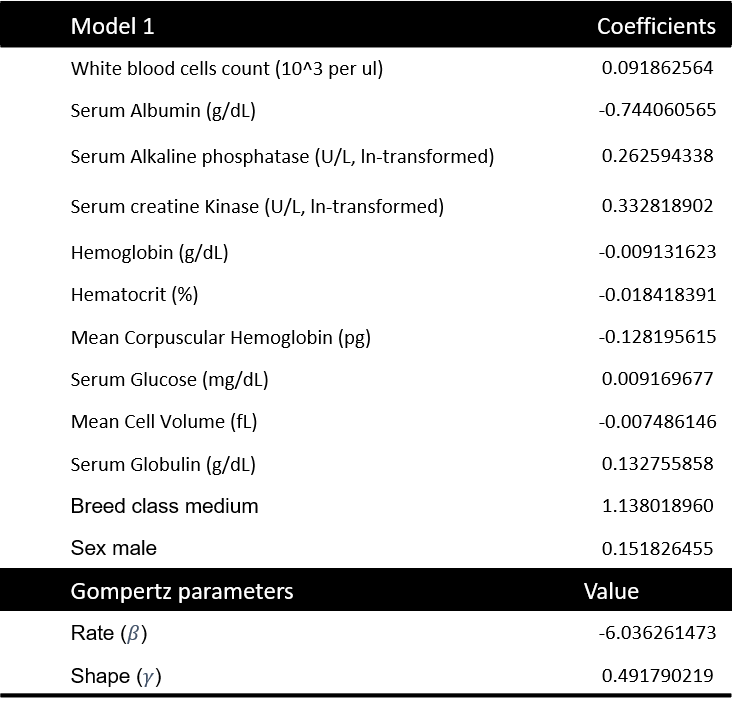


Dog p$\mathrm{henoage}=$ $\ln\left( \frac{\gamma_{0}*e^{xb}*\{e^{\gamma*age}- 1\}}{e^{\left\{ breed*\beta_{breed} \right\}+\left\{ sex * \beta_{sex} \right\}+ \beta_{0}}* \gamma}+1 \right) *\frac{1}{\gamma_{0}}$

$xb= \beta$ *+* $\sum(coef *value)$


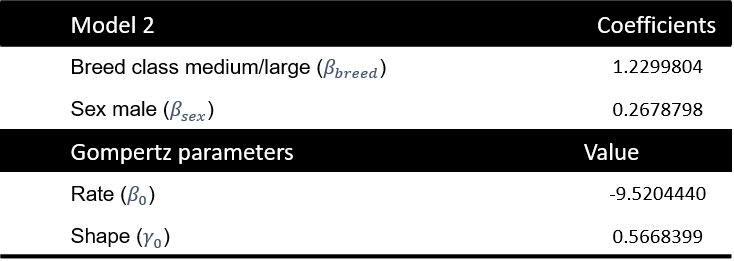


a

b

**Fig S3**

**a:** Gompertz coefficients for the dog Biological Age. **B**: Biological Age formula.


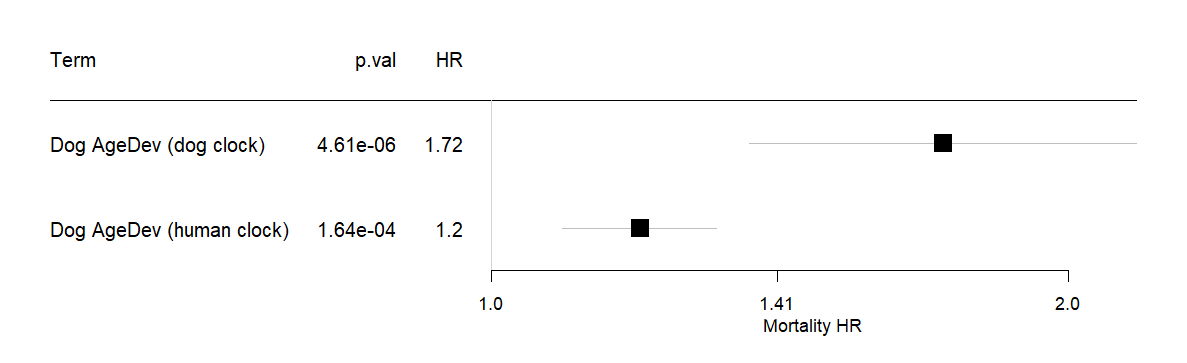


**Fig S4**

Analyzing the survival prediction on the dog data set with a dog-specific phenoage algorithm (developed in this manuscript) compared to the published human phenoage algorithm (truncated for parameters that are not available in routine canine laboratory analysis). HR: hazard ratio. Data indicate HR with 95% confidence intervals using cox proportional hazard model for all-cause mortality.

**
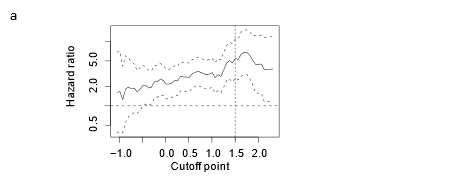
**

**Fig S5**

Evaluation of hazard ratio when AgeDev is treated as a binary outcome at different cut-off points along the range of observed values (-1 to 2).


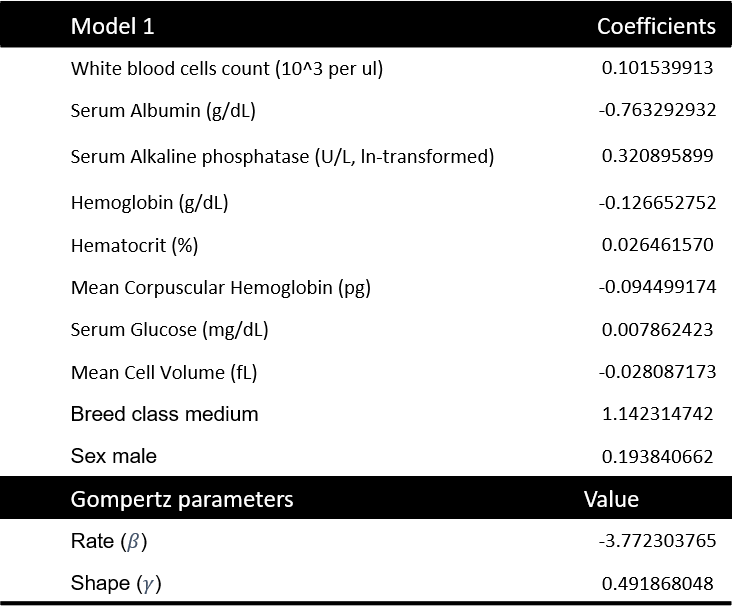

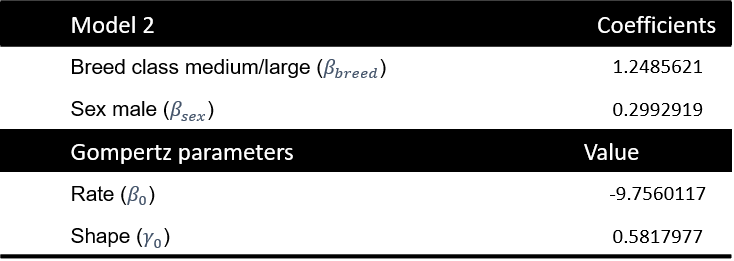


**Fig S6**

**a:** Gompertz coefficients for the reduced Biological Age model with 8 parameters.


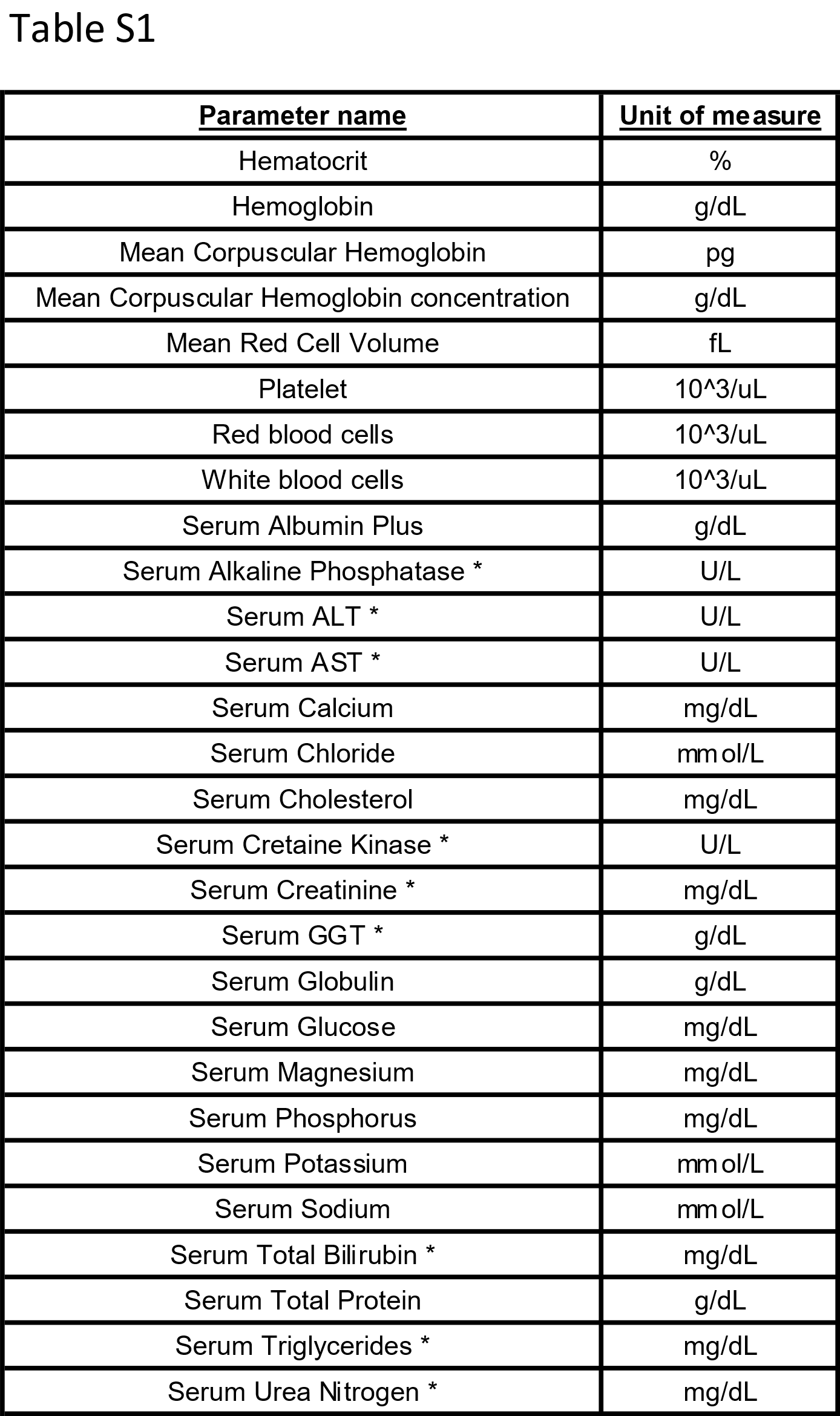


**Table S1**

List of parameters routinely measured in the petcare center that entered into the penalized Cox regression, along with unit of measure. Asterisks indicate parameters that were log-transformed before analysis using natural log.
